# Retraction Note to: Restoring microRNA-499-5p Protects Sepsis-Induced Lung Injury Mice Via Targeting Sox6

**DOI:** 10.1186/s11671-022-03725-0

**Published:** 2022-09-06

**Authors:** Wenjie Zhang, Jing Li, Hui Yao, Tianmin Li

**Affiliations:** 1 Intensive Care Unit (ICU), Weihai Municipal Hospital, Cheeloo College of Medicine, Shandong University, No. 70, Heping Road, Weihai, 264200 Shandong China; 2 Preventive Medicine Ward, Weihai Municipal Hospital, Cheeloo College of Medicine, Shandong University, Weihai, 264200 Shandong China

## Retraction: Nanoscale Research Letters (2021) 16:89 10.1186/s11671-021-03534-x

The Editors-in-Chief have retracted this article. After publication, the authors requested a retraction because they were unable to reproduce some of the results. They also stated that they had failed to obtain ethics approval for experiments involving animals. Additional concerns were raised about the quality of Western blots presented in the article, but the authors did not respond to the request to provide raw data.

The Editors-in-Chief therefore no longer have confidence in the integrity of the data in this article. The authors did not respond to any correspondence from the editor about the wording of this retraction notice.

